# Prevalence of Microvascular Complications in Newly Diagnosed Patients with Type 2 Diabetes 

**DOI:** 10.12669/pjms.294.3704

**Published:** 2013

**Authors:** Alia Ali, Farrukh Iqbal, Azeem Taj, Zafar Iqbal, Muhammad Joher Amin, Qasim Zafar Iqbal

**Affiliations:** 1 Alia Ali, FCPS, Senior Registrar, Department of Medicine, Shaikh Zayed Postgraduate Medical Institute, Lahore, Pakistan.; 2 Farrukh Iqbal, FRCP, Professor, Department of Medicine, Shaikh Zayed Postgraduate Medical Institute, Lahore, Pakistan.; 3 Azeem Taj, FCPS, Associate Professor, Department of Medicine, Shaikh Zayed Postgraduate Medical Institute, Lahore, Pakistan.; 4 Zafar Iqbal, FRCP, Professor, Department of Medicine, Shaikh Zayed Postgraduate Medical Institute, Lahore, Pakistan.; 5 Muhammad Joher Amin, FCPS, Assistant Professor of Gastroenterology, Shaikh Zayed Postgraduate Medical Institute, Lahore, Pakistan. Department of Medicine, Shaikh Zayed Postgraduate Medical Institute, Lahore, Pakistan.; 6 Qasim Zafar Iqbal, MBBS (Student), Shaikh Zayed Hospital, Lahore, Pakistan. Department of Medicine, Shaikh Zayed Postgraduate Medical Institute, Lahore, Pakistan.

**Keywords:** Microvascular Diabetic Complications, Neuropathy, Nephropathy, Retinopathy, Type 2 Diabetes Mellitus

## Abstract

***Background & Objective:*** Microvascular complications are the major outcome of type 2 Diabetes Mellitus progression, which reduce the quality of life, incur heavy economic burdens to the health care system and increase diabetic mortality. The aims of this study were to assess the prevalence of microvascular complications among newly diagnosed type 2 diabetic patients and to analyze the association between these complications and poor glycemic control.

***Methods:*** This cross sectional hospital based study was carried out in Diabetic Clinic of Shaikh Zayed Postgraduate Medical Institute, Lahore Pakistan. The study was conducted from November 2011 to November 2012 among newly diagnosed type 2 diabetic patients. Relevant information of all patients was recorded with the help of a proforma. They were investigated for retinopathy, nephropathy and neuropathy.

***Results:*** We have divided the patients into two groups: Group I with good glycemic control (HbA_1_c <6.5) and group II with poor glycemic control (HbA_1_c >6.5). In group II microvascular complications were 89.8%. Neuropathy, nephropathy and retinopathy were present in 68.5%, 56.2% and 31.4% respectively. These similar percentages in Group I were 50%, 0% and 31% respectively and are significantly lower.

***Conclusion: ***The study showed that even in newly diagnosed type 2 diabetic patients who had poor glycemic control, frequency of microvascular complications is much higher as compared to those who had average glycemic control. Thus tight glycemic control does count even in newly diagnosed type 2 diabetics to prevent and minimize the occurrence of complications.

## INTRODUCTION

Diabetes mellitus is a global epidemic that affects more than 150 million people worldwide.^1^ It is estimated that global number of adults suffering from any form of diabetes will reach 439 million in 2030; most of them type 2 diabetes mellitus cases.^2^^,^^3^Diabetes mellitus is a major cause of morbidity and mortality. Data from prospective and cross-sectional studies consistently points to the fact that diabetic patients are more likely to develop micro- as well as macrovascular conditions.^4^^,^^5^

Microvascular complications from type 2 diabetes are common and include retinopathy leading to various degrees of visual impairment including blindness and has become a major cause of blindness throughout the world^1^^,^^6^; neuropathy, leading to pain and numbness, chronic and recurrent infected ulcers in the extremities which can lead to amputation; and nephropathy characterized by proteinuria ultimately leading to end stage renal disease. It constitutes the major work load of dialysis centers.^3^^,^^7^

It is well known that these chronic complications are the major outcome of type 2 diabetes mellitus progress, which reduces the quality of life of patients, incur heavy burdens to the health care systems, and increase diabetic mortality.^8^^,^^9^Several large clinical trials have demonstrated that tight blood glucose control correlates with a reduction in the microvascular complications of diabetes.^10^The American Diabetes Association (ADA) has designated HbA1C level of <7% as a goal of optimal blood glucose control^11^ and the American Association of clinical endocrinologists has further recommended HbA1C levels of <6.5%.^12^While it has not always been clear that aggressive glycemic control can reduce the end-organ complications of diabetes^13^, recent evidence indicates that aggressive glycemic control in type 2 diabetes is associated with a 25% lower incidence of microvascular end points.^14^^,^^15^

Access to diabetic care is limited in low and middle income countries (including Pakistan) where more than 70% of diabetic patients live.^16^ As a transitional society, Pakistan is facing a rapid rise of type 2 diabetic population accompanying its remarkable economic development, especially **in** urban population. Yet, few studies have addressed the extent of the type 2 diabetes mellitus epidemic as well as the disease burden of diabetic complications to Pakistan’s health care system.

It is obvious that information on prevalence of type 2 diabetes mellitus related complications is important for the adjustment of policies and practices in diabetic care management to gain better control of type 2 diabetes mellitus.

The aims of this study were to determine the frequency of microvascular complications in newly diagnosed type 2 diabetic patients in association with glycemic control.

## METHODS

A total of one hundred and thirteen (113) newly diagnosed diabetic patients who attended the diabetic clinic of Sheikh Zayed Postgraduate Medical Institute, Lahore Pakistan, were included in the study. Newly diagnosed type 2 diabetics are defined as those type 2 diabetic patients who were presented to us within 6 months of their diagnosis of Diabetes Mellitus. These patients were diagnosed as having DM on the basis of either a random plasma glucose level of 200mg/dl(11.1 mmol/L) or higher together with classical features of DM such as polyuria, polydipsia, polyphagia and weight loss or a fasting blood sugar(FBS) level of >126mg/dl(7.0mmol/L) or higher or Haemoglobin A1c( HbA1c) level of >6.5% or higher.The study was conducted during the period of one year from November 2011 to November 2012.

Patients’ age ranges from 30-70 years. Informed consent was obtained from each patient. A structured questionnaire regarding the demographic data such as age, sex, duration of diabetes, height and body weight were measured while wearing light weight clothing, without shoes. Blood pressure, smoking habit, family history of diabetes and hypertension were recorded for each patient. Diabetic patients suffering from any other medical problems were excluded from the study. Co-morbidities like chronic heart, renal and liver diseases as evidenced by either history of ischemic heart disease or alteration in ECG; history of renal disease or disturbed BUN, creatinine; history of liver disease such as hepatitis B or C positive or disturbed liver function tests respectively. BMI was calculated as weight (kg) divided by height (m2).

Blood samples were obtained for HbA1c test that was estimated using chromatographic method by antiticalcon kit from USA. All assays were performed as per instructions of the manufactures.

***Retinopathy: ***Patients with bilateral cataracts were excluded for retinopathy. Retinopathy assessed by direct ophthalmoscope that was done after pupillary dilatation by tropicamide 1% eye drops was defined as the presence of at least one micro aneurysm or hemorrhage or exudates in either of the eye.^17^

***Neuropathy: ***Neuropathy was diagnosed by history of numbness, paraesthesias, tingling sensations, burning sensation and confirmed by touch sensation using 10gm monofilament, vibration sense by tuning fork (128 Hz) and ankle reflex. Painful peripheral neuropathy was diagnosed by history of pain worsening at night Autonomic neuropathy was diagnosed by history of constipation or diarrhea, gastroparesis and postural hypotension confirmed by blood pressure recording in lying down and standing positions.

***Nephropathy: ***Morning midstream urine sample, negative for proteinuria by Albustix was used to calculate micro albumin: creatinine ratio in mg/g. Microalbumin was carried out using ELISA assay developed at NHRC and validated at NETRIA UK, where as creatinine was done by colorimetric methods using Fortress kit USA. If the ratio was <30mg/g the patient was normoalbuminuric, ratios between 30-300mg/g were indicative of microalbuminuria and above 300mg/g revealed macroalbuminuria. 

Subjects were excluded from the study if they came to the clinic after vigorous exercise, had any serious illness such as history of heart failure, UTI or were known patients of nephropathy. Statistical analysis was done using SPSS version 20.

## RESULTS

One hundred and thirteen newly diagnosed type 2 diabetic patients attending the diabetic clinic of the Shaikh Zayed postgraduate medical institute Lahore were considered for the study. The study was conducted over the period of one year from November 2011 to November 2012. Patients were divided into two groups as assessed by mean of three consecutive HbA1c levels, either <6.5 or >6.5.

**Group I: **(n=24) were those patients with optimal glycemic control (mean HbA1c <6.5).

**Group II:** (n=89) were those patients with poor glycemic control (mean HbA1c ≥ 6.5).

Baseline characteristics in two groups: The mean (±SD) age in group I was 48.04±8.38 years while in group II was 49.55±9.6 years. Gender distribution in group I showed 8 males and 16 females while in group II, 31 males and 58 females. In group I, mean BMI was 29.042±3.44 while in group II it was 28.702±4.30.

Family history in group I was positive in 50% while in group II it was positive in 78.6% patients. These results showed no significant statistical difference between the baseline characteristics of two groups (Table-I).

**Table-I:** Baseline Characteristics of Two Groups

	*Group 1* *(n=24)*	*Group 2* *(n=89)*	*Remarks*
Age (mean)	48.04± 8.38	49.55± 9.6	P=0.48
Gender Male Female	8 16	31 58	p=0.547
BMI (mean±SD)	29.042±3.44	28.702±4.30	p value=0.7
Family History Positive	7	29	p value=0.8

***Microvascular complications*** *: *The two groups were assessed for microvascular complications such as neuropathy, nephropathy and retinopathy (Table-II). Any of the microvascular complications in group I were 50% while in group II were 89.8%. These results showed significant statistical difference (p<0.001). 

Neuropathy in group I was present in 50% of patients while in group II it was present in 68.5% patients. Though the percentage was higher in group II but the results did not show statistically significant difference (p = .076). None of the patients had nephropathy in group I, while in group II it was present in 56.1% patients and the results showed statistically significant difference (p<0.0001). Retinopathy was present in 0.3% patients in group I while 31.4% patients in group II and again results were statistically highly significant (p<0.001).

Group II patients showed much higher percentage of microvascular complications in combination as well as separately with significant statistical difference as compared to group I as shown in Table-II.

**Table-II:** Comparison between two groups regarding microvascular complications

	*Group 1* *N=24*	*Group 2* *N=89*	*p value*
Microvascular Complications	12	80	<0.001
Neuropathy	12	61	0.076
Microalbuminuria	0	50	<0.001
Retinopathy	2	28	0.017

## DISCUSSION

Diabetes Mellitus is the commonest metabolic disorder. It is growing world wide like an epidemic and our population is not an exception that is why it has high prevalence in Pakistan. Moreover being inhabitants of a developing country, we are at increased risk of contracting this disease.^18^

It is characterized by microvascular and macrovascular complications that substantially increase the morbidity and mortality associated with the disease and reduces the quality of life. Hyperglycemia is an important risk factor for the development of microvascualr disease in patients with type-2 diabetes, as it is in patients with type-1 DM. This has been shown in several observational studies.^19^,^20^ A landmark study on type 2 diabetes, United Kingdom Prospective Diabetes Study (UKPDS)^21^ also showed that the risk for occurrence of microvascular and macrovascular complication was shown to increase at HbA1c levels of 6.5% or more (i.e. poor glycemic control). Poor glycemic control in type-2 diabetes has serious implications on health and is a major risk factor for the development of diabetic complications.^22^

Our study also showed strong correlation between poor glycemic control and microvascular complications even in newly diagnosed type-2 diabetics. In our study 78.76% of total newly diagnosed type-2 diabetics showed poor glycemic control (i.e. Hb1c >6.5) which is strongly associated with increased frequency of microvascular complications (n=90%). This percentage is much higher in our study as compared to other studies.^23^-^25^ Similarly if we see the percentage of microvascular complications separately, still it is higher among patients with poor glycemic control showing 68.5% neuropathy, 56.1% nephropathy and 31.4% of retinopathy respectively. (Table II).

Interestingly, none of the patient had nephropathy with good glycemic control.^25^ Gupta et al^25^ from New Delhi also found that glycosylated Hb was significantly higher in microalbuminuric type-2 diabetic patients. Similarly as we observed that poor glycemic control was associated with increased incidence of diabetic retinopathy and these results were consistent with findings of Rema et al^23^, Klein R et al^24^ and Knuiman et al.^26^ In the same way we have observed that poor diabetic control was the most important factor related to diabetic neuropathy as shown in other studies.^23^^.^^24^ In our study we have also observed that new onset diabetes is no longer safe from developing microvascular complications as we have seen that even in newly diagnosed type-2 patients the incidence of these complications is much higher i.e. 78.76%.

This factor added to the fact from other studies like Shera et al^27^ from Pakistan had shown that microvascular complications were significantly related to the duration of diabetes. Agarwal et al India also observed that the duration of diabetes has an influence on retinopathy, peripheral vascular disease, neuropathy and nephropathy.^28^

Thus our study revealed that even in newly diagnosed type-2 patients with poor glycemic control, tight or at least optimal glycemic control does count a lot to prevent the occurrence of early microvascular complications.

***Limitations of the study:*** It included small sample size, hospital based study and absence of control group.

## CONCLUSION

It is concluded that even in newly diagnosed patients with diabetes poor glycemic control is responsible for increased microvascular complications. So effective screening method and adequate control of diabetes should be done to address this issue. Newly diagnosed type-2 diabetic patients with poor glycemic control, referred from primary care to Diabetic clinics should be strongly encouraged for optimal or even (good) tight glycemic control in order to prevent early emergence of microvascular complications that leads to increased morbidity and mortality in these patients.
